# Texture Analysis as Imaging Biomarker for recurrence in advanced cervical cancer treated with CCRT

**DOI:** 10.1038/s41598-018-29838-0

**Published:** 2018-07-30

**Authors:** Jie Meng, Shunli Liu, Lijing Zhu, Li Zhu, Huanhuan Wang, Li Xie, Yue Guan, Jian He, Xiaofeng Yang, Zhengyang Zhou

**Affiliations:** 1 Department of Radiology, Nanjing Drum Tower Hospital, The Affiliated Hospital of Nanjing University Medical School, 210008 Nanjing, China; 2 The Comprehensive Cancer Centre of Drum Tower Hospital, The Affiliated Hospital of Nanjing University Medical School, China Nanjing, 210008; 3 School of Electronic Science and Engineering, Nanjing University, 210046 Nanjing, China; 4 Department of Radiation Oncology and Winship Cancer Institute, Emory University, Atlanta, Georgia 30322 USA

## Abstract

This prospective study explored the application of texture features extracted from T2WI and apparent diffusion coefficient (ADC) maps in predicting recurrence of advanced cervical cancer patients treated with concurrent chemoradiotherapy (CCRT). We included 34 patients with advanced cervical cancer who underwent pelvic MR imaging before, during and after CCRT. Radiomic feature extraction was performed by using software at T2WI and ADC maps. The performance of texture parameters in predicting recurrence was evaluated. After a median follow-up of 31 months, eleven patients (32.4%) had recurrence. At four weeks after CCRT initiated, the most textural parameters (four T2 textural parameters and two ADC textural parameters) showed significant difference between the recurrence and nonrecurrence group (P values range, 0.002~0.046). Among them, RunLengthNonuniformity (RLN) from T2 and energy from ADC maps were the best selected predictors and together yield an AUC of 0.885. The support vector machine (SVM) classifier using ADC textural parameters performed best in predicting recurrence, while combining T2 textural parameters may add little value in prognosis. T2 and ADC textural parameters have potential as non-invasive imaging biomarkers in early predicting recurrence in advanced cervical cancer treated with CCRT.

## Introduction

Cervical cancer is the fourth leading cause of cancer death in females worldwide. Concurrent chemoradiotherapy (CCRT) is the standard treatment for locally advanced cervical cancer. However, approximately one third of patients would experience recurrence^1,2^. By using tumor morphology-based response criteria, tumor recurrence is frequently not detected until many months after the completion of primary therapy. The heterogeneous therapy responsiveness and the dilemma in reliably predicting the long-term treatment outcome presents a major challenge for developing a more precise personalized care^3^. If there are reliable biomarkers that can early identify patients who are at high risk of recurrence, clinicians could adjust treatment regimen (such as dose escalation or addition of adjuvant therapies) in time for those patients.

As a noninvasive functional imaging technique, diffusion weighted imaging (DWI) has been widely used in the prediction of treatment outcome in cervical cancer research but the accuracy is limited. For example, there were conflicting reports about whether pretreatment apparent diffusion coefficient (ADC) related parameters had prognostic value^4,5^. There is emerging evidence that decreases in tumor heterogeneity generally associated with improved outcomes^6^. However, previous imaging prognostic biomarkers were usually derived from mean values or simple histogram analysis, which were insufficient for assessing intratumor spatial heterogeneity^7,8^.

Texture analysis refers to a variety of mathematical methods that can evaluate the gray-level intensity and position of the pixels within an image. It generates a range of quantitative imaging features so-called ‘texture features’ that provide a measure of intralesional heterogeneity^9^. Texture analysis has been applied to computed tomography (CT), magnetic resonance imaging (MRI) and positron emission tomography (PET) studies. It was reported that some pretreatment texture features as well as changes of texture features were associated with treatment outcome in various tumors^10–13^. To date, there have been a few reports on cervical cancer prognosis using texture analysis. Sylvain *et al*. and Ho *et al*. found texture features extracted from PET images could predict recurrence of cervical cancer better than SUV_max_ while PET is less clinically used than MRI with radiation exposure^14,15^. Jeffrey *et al*. reported that texture features based on dynamic contrast-enhanced MRI (DCE-MRI) performed well in prediction recurrence but with a very limited sample size of 23^16^. Most studies on MR texture analysis used functional imaging such as DCE and DWI to obtain texture features, attentions have also been paid to the application to routine T1- and T2- weighted images (T2WI)^17,18^.

It is known that the ADC values are affected by perfusion, diffusion factors and artifacts. Furthermore, DCE MR imaging uses contrast media containing gadolinium which could induce nephrogenic systemic fibrosis in patients. Thus, recently, texture analysis based on T2WI have been investigated in oncologic imaging by several groups. Vignati *et al*. found that some texture features calculated on T2WI outperform ADC parameters in predicting prostate cancer aggressiveness^19^. A recent study reported that texture features were associated with pathologic complete response only at T2WI but not at DCE in breast cancer treated with neoadjuvant chemotherapy^20^. Carlo *et al*. demonstrated the efficacy of using texture analysis based on T2WI to predict tumor response to neoadjuvant chemoradiotherapy in rectal cancer^21^. To the best of our knowledge, there have been no previous reports examining texture analysis based on routine T2WI or DWI sequences for the prognosis of cervical cancer.

In the present study, we aimed to explore more promising texture features extracted from pre- and post-treatment T2WI and ADC maps to non-invasively predict recurrence of advanced cervical cancer patients treated with CCRT.

## Results

### Follow-up outcome

**Figure 1:**
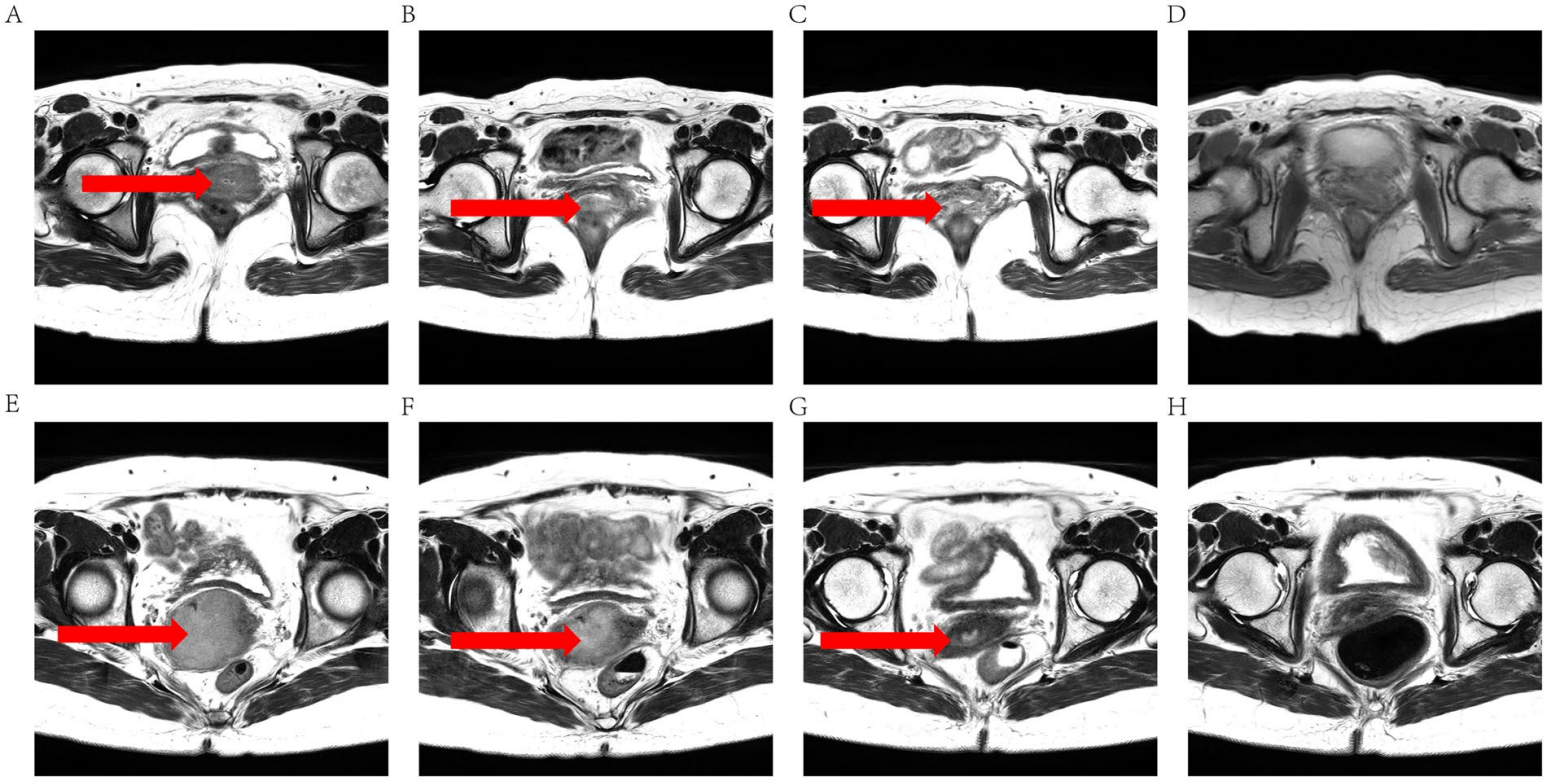
T2-weighted MR images of two representative patients with cervical cancer during the course of concurrent chemoradiotherapy (CCRT). (**A**–**D**) a 46-year-old woman with cervical cancer (the international federation of gynecology and obstetrics FIGO stage, IIB) who had recurrence 8 months after CCRT completed. (E-H) a 59-year-old woman with cervical cancer (FIGO stage, IIIB) who maintained complete response during follow-up. (**A**,**E**) before CCRT, tumor of the recurrence case is smaller than tumor of the nonrecurrence case; (**B**,**F**) 2 weeks after CCRT initiated, tumor shows a significant decrease in size in both cases; (**C**,**G**) 4 weeks after CCRT initiated, tumor continues to shrink in both cases; (**D**,**H**) one month after CCRT completion, no obvious residual lesions could be seen on T2w images of both cases. Those two representative cases illustrate the difficulty of predicting cervical cancer recurrence for clinicians.

Among the 34 patients, 23 patients (23/34, 67.6%; mean age, 47.2 years) showed nonrecurrence and the remaining 11 patients (11/34, 32.4%; mean age, 54.7 years) were classified as recurrence group (4 deaths, 5 local recurrence, and 2 disease progression). Two representative cases of cervical cancer with different long-term prognosis illustrate the difficulty of predicting recurrence for clinicians (Fig. 1).

### Texture parameters between different prognosis groups

At timepoint 1, Only several T2 textual parameters including 5 Percentile, range and RLN showed significant difference between the recurrence and nonrecurrence groups. (P values: 0.004, 0.008, 0.015).

At timepoint 2, T2 textual parameter RLN and ADC textural parameter correlation-GLCM25 were significant different between groups. (P values: 0.023; 0.011).

At timepoint 3, T2 textual parameters 5 Percentile, RLN, GaussAmplitude, contrast-NIDM and ADC textural parameters correlation-GLCM25, energy showed significant difference between groups. (P values: 0.039, 0.002, 0.044, 0.003; 0.034, 0.002).

At timepoint 4, only two ADC textural parameters LRHGE and GaussAmplitude were significant different between groups. (P values: 0.034, 0.046).

**Figure 2:**
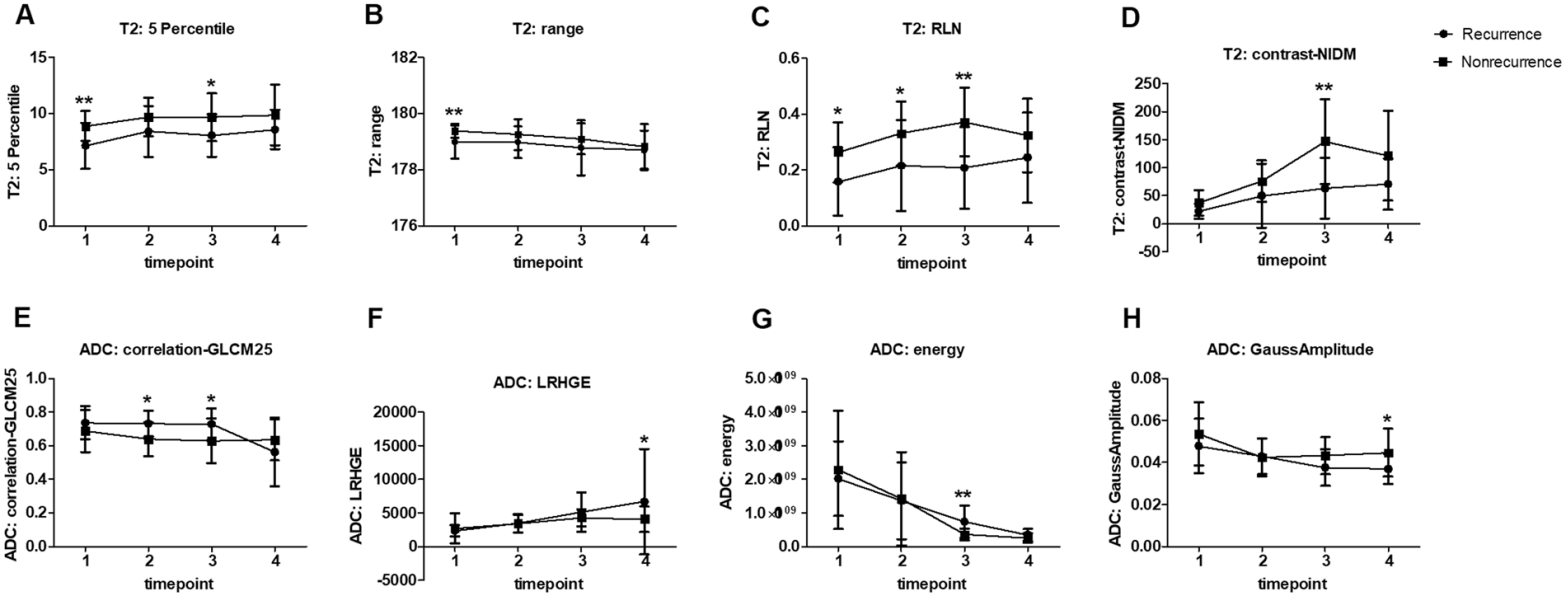
The variety trends of T2 and ADC textural parameters that can differentiate the recurrence and nonrecurrence groups in cervical cancers underwent concurrent chemoradiotherapy (CCRT). (**A**–**D**) T2 textural parameters 5 Percentile (at timepoint 1 and 3), range (at timepoint 1), RLN (at timepoint 1, 2 and 3) and contrast-NIDM (at timepoint 3) in the recurrence group was significantly lower than those in the nonrecurrence group. (**B**–**H**) ADC textural parameters correlation-CLCM25 (at timepoint 2 and 3), LRHGE (at timepoint 4), energy (at timepoint 3) and GaussAmplitude (at timepoint 4) showed significant difference between groups. Timepoint 1: before CCRT; timepoint 2: 2 weeks after CCRT initiated; timepoint 3: 4 weeks after CCRT initiated; timepoint 4: one month after CCRT completion. ^*^P < 0.05; ^**^P < 0.01.

The variety trends of textural parameters that can differentiate the recurrence and nonrecurrence groups are shown in Fig. 2.

### Logistic regression models for predicting recurrence

At timepoint 2, with the best selected predictor as RLN from T2WI, the AUC for predicting recurrence was 0.739.

**Figure 3:**
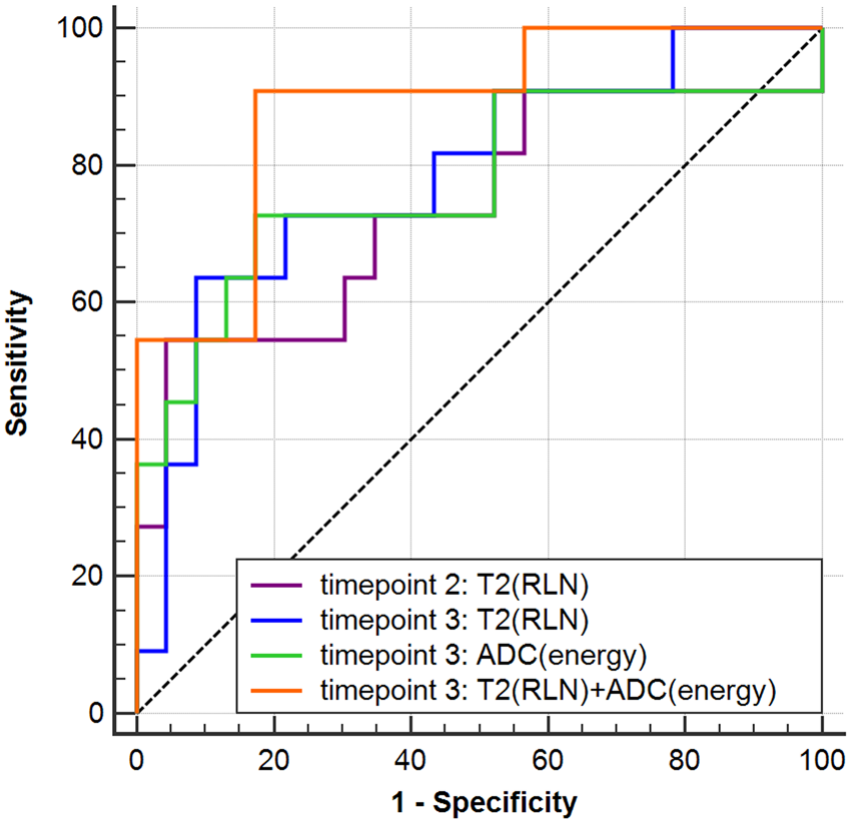
The Receiver operating characteristic (ROC) curves of logistic regression models for predicting recurrence in advanced cervical cancer treated with concurrent chemoradiotherapy (CCRT). At timepoint 2, T2 textural parameter RLN yield an area under the curve (AUC) of 0.739. At timepoint 3, T2 textural parameter RLN yield an AUC of 0.787, while ADC textural parameter energy yield an AUC of 0.775, and their combination improved the AUC to 0.885. Timepoint 2: 2 weeks after CCRT initiated; timepoint 3: 4 weeks after CCRT initiated.

At timepoint 3, for T2 texture analysis, RLN was the best selected predictor and yield an AUC as 0.787. For ADC texture analysis, energy was the best selected predictor and yield an AUC as 0.775. After combining all parameters from T2WI and ADC maps, with the best selected predictors as RLN from T2WI and energy from ADC maps, the AUC could be improved to 0.885, though there were no significant differences between any two AUCs of the features (all P values > 0.05). The ROC curves of each regression model in predicting recurrence are displayed in Fig. 3. The detailed performances of each regression model are shown in Table 1.

**Table 1:** The performance of each regression model for predicting recurrence in cervical cancer treated with chemoradiotherapy (CCRT).

	AUC	P	Sensitivity	Specificity	Accuracy
Timepoint 2:					
T2-RLN	0.739	0.026	54.55	95.65	82.35
Timepoint 3:					
T2-RLN	0.787	0.008	63.64	91.30	82.35
ADC-energy	0.775	0.011	72.73	82.61	79.43
T2-RLN + ADC-energy	0.885	<0.001	90.91	82.61	85.30

Note: T2-RLN represents RunLengthNonuniformity (RLN) from T2WI; ADC-energy represents energy from apparent diffusion coefficient (ADC) maps Timepoint 2: two weeks after CCRT initiated; Timepoint 3: four weeks after CCRT initiated.

At timepoint 1 and timepoint 4, no parameter was selected in the regression model.

### Results of supervised classification

Since at timepoint 3, the number of parameters that could differentiate different groups were the most, and the regression models at timepoint 3 show relatively high AUCs for predicting recurrence, we considered timepoint 3 as the best timepoint for early predicting recurrence. The SVM classification results obtained by cross-validation on T2, ADC and T2 + ADC textural parameters at timepoint 3 are shown in Table 2. Textural parameters extracted from ADC maps had higher accuracy, sensitivity and specificity than those extracted from T2WI. T2 + ADC textural parameters also performed well in predicting recurrence, but did not show obviously better results than the sole ADC textual parameters.

**Table 2:** The performance of the support vector machine (SVM) classification obtained by cross-validation for predicting cervical cancer recurrence at four weeks after chemoradiotherapy initiated.

Imaging	Dataset	Sensitivity	Specificity	Accuracy	PPV	NPV	AUC
T2WI	Training	0.80	0.85	0.83	0.71	0.91	0.83
	Testing	0.46	0.76	0.65	0.60	0.71	0.61
ADC	Training	1.00	1.00	1.00	1.00	1.00	0.98
	Testing	0.47	0.87	0.71	0.63	0.74	0.74
T2WI + ADC	Training	0.90	0.91	0.91	0.84	0.96	0.94
	Testing	0.47	0.80	0.68	0.61	0.73	0.73

Note: PPV = positive predictive value; NPV = negative predictive value; AUC = area under the curve; ADC = apparent diffusion coefficient.

## Discussion

The results of our study demonstrate the potential use of texture analysis based on T2WI and ADC maps to predict recurrence of advanced cervical cancer treated with CCRT. We also found that four weeks after CCRT initiated was the optimal timepoint for early predicting cervical cancer recurrence. ADC textural parameters at four weeks after CCRT initiated performed best in predicting recurrence, while combining T2 textural parameters may add little value in prognosis.

Preliminary reports have hinted at the potential use of texture analysis in cervical cancer imaging. Becker *et al*. reported that ADC textural parameter LRHGE correlated with the differentiation of cervical cancer^22^. Ho *et al*. applied PET texture analysis to cervical cancer prognosis and found RLN as one of good predictors of post-CCRT complete metabolic response with an AUC of 0.75^15^. Our study had several important differences compared with the existing literature. Most previous studies applying texture analysis to tumor prognosis only focused on one or two timepoints^15,21,23^. In the current work, intratumoral heterogeneity depicted by texture parameters was evaluated at four different timepoints including baseline, 2nd week and 4th week during therapy and after the completion of treatment. Hence, we can not only explore the temporal behaviors of tumor heterogeneity, but also find which timepoint was optimal for predicting recurrence in advanced cervical cancer treated with CCRT. Our study found timepoint 3 was the best timepoint for two main reasons: firstly, at timepoint 3, the number of texture parameters that could differentiate different groups were the most, and the regression models at timepoint 3 show relatively high AUCs for predicting recurrence. Secondly, pre-treatment texture parameters at timepoint 1 can only represent the inherent heterogeneity characteristics of the tumor, while mid-treatment texture parameters also reflect tumor microenvironment change information caused by anti-cancer treatment. At timepoint 3, the tumor microenvironment changes are greater than that at timepoint 2, thus may better predict tumor response to the treatment and long-term prognosis.

During CCRT, textural parameters 5 Percentile, RLN, contrast-NIDM and LRHGE showed ascending temporal trends while range, correlation-GLCM25 and energy showed descending temporal trends. These changing trends indicated that tumor heterogeneity reduced after treatment. However, no obvious difference between the general variety trends of the recurrence and nonrecurrence groups was observed in this study. Logistic regression analysis selected the most discriminatory two features textural parameters, namely RLN derived from T2WI and energy derived from ADC maps at timepoint 3. The SVM classification results also indicated timepoint 3 as a good timepoint for early predicting recurrence. However, some previous studies demonstrated that baseline MR textual parameters could have potential in predicting treatment response in breast cancer^20^, rectal cancer^21^ and glioblastoma^24^. Although we found several baseline T2 textural parameters including 5 Percentile, range and RLN in the recurrence group were significantly lower than those in the nonrecurrence group, none of  baseline textural parameters was selected in the regression model. The possible explanation may be that pre-treatment MRI data can only reflect inherent intratumoral heterogeneity information, while post-treatment MRI data represent the current status of the tumor after chemoradiotherapy.

The prognostic model in our study combined both T2WI and DWI data of patients with advanced cervical cancer. This combination method has been used in the diagnosis and grading of prostate cancer^19,25^ as well as the prognosis of rectal cancer^26^, but so far it has not been reported for assessing cervical cancer. We found combination of T2 textural parameter RLN and ADC textural parameter energy yield a little higher AUC (0.885) than either of them alone (AUC = 0.787, 0.775, respectively). And the SVM classification showed that combining T2 textural parameters may add little value in prognosis. We speculated that morphological features from T2WI reflect only limited information about residual tumor posttreatment, DWI may provide more valuable details regarding the response to CCRT in advanced cervical cancer. A recent study by Liu *et al*. also confirmed this. They constructed a radiomics signature for pCR assessment after chemoradiotherapy in rectal cancer, and found that only 1 T2WI feature was selected in the model while the others were all ADC features^26^.

Another advantage of our study was that our measurement for heterogeneity on T2WI and ADC maps was done at all slices covering the whole tumor. The whole-tumor texture analysis is more representative of tumor heterogeneity than some previous studies using the single largest cross-sectional area analysis^23^. What’s more, to strengthen our study, the MR imaging technique was standardized and uniform across the study population.

Our study also had some limitations. Firstly, the number of patients in this preliminary study was still limited. As a result, when performing SVM classification, the training and testing were performed on the same set of patient data. To minimize the bias, the patient data were stratified sampled with 70% of them used for training while the remaining 30% of them used for testing purpose. All the tests were run 100 times with the average value reported as the cross-validated performance. Study with larger sample size as well as an external validation are required to confirm the prognostic performance of these textural parameters. Secondly, we visually showed changing trends of some representative textural parameters, but did not investigate their change rates, which may also be related to prognosis. Studies on correlation between textural parameters change rates and tumor recurrence is needful in the future. Thirdly, the follow-up was not long enough to assess the predictive value on patient survival. A longitudinal study is needed to further understand the long-term prognostic value of MR texture analysis in cervical cancer.

## Conclusion

Our study suggests the potential of T2 and ADC textural parameters as non-invasive imaging biomarkers in early predicting recurrence in advanced cervical cancer treated with CCRT, which may provide an opportunity for clinicians to adjust therapeutic strategies in time to develop a more individualized anti-cancer treatment.

## Materials and Methods

### Patient cohort

**Figure 4:**
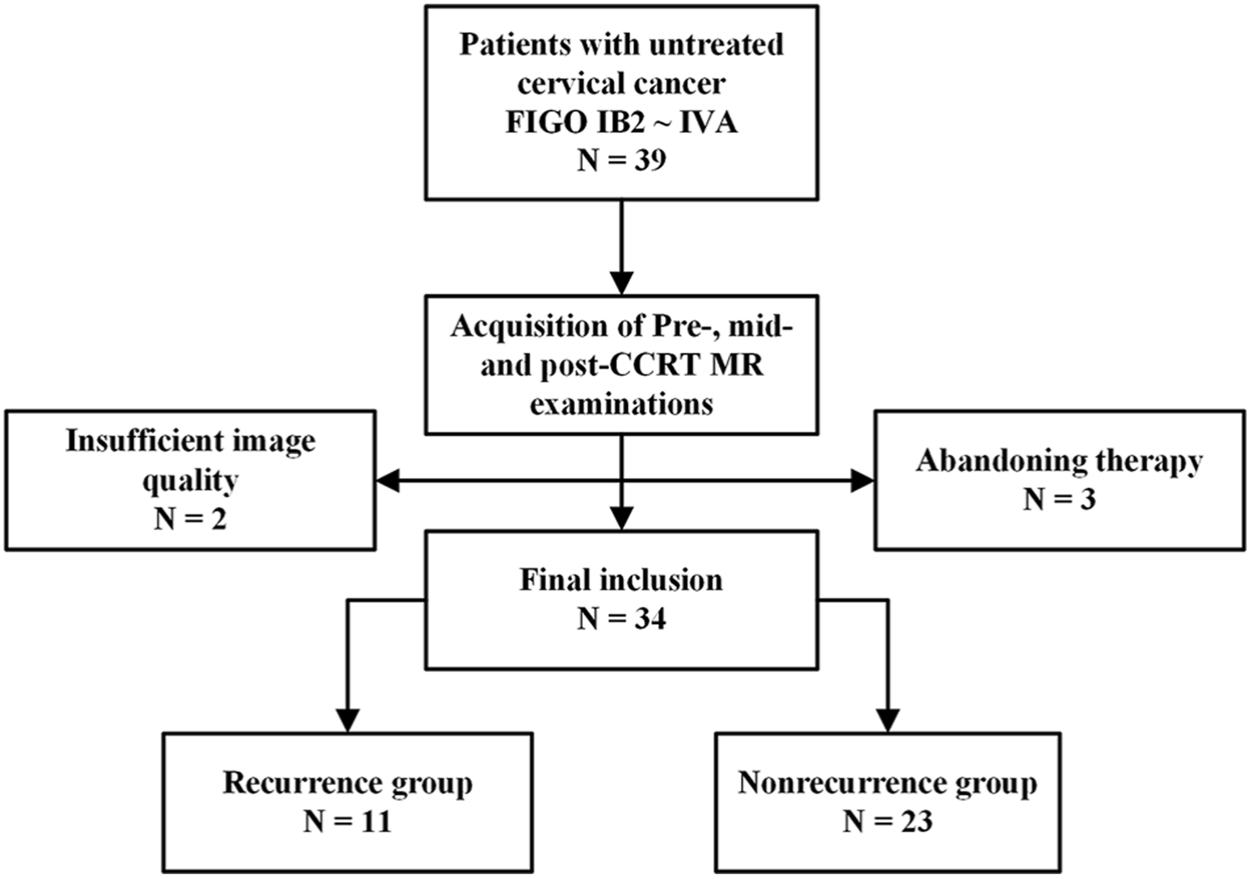
Flowchart of the study population. FIGO = International Federation of Gynecology and Obstetrics, CCRT = concurrent chemo-radiotherapy.

This study was approved by the ethics committee of the Institutional Review Board of Nanjing Drum Tower Hospital, and written informed consent was obtained from all patients. The methods were carried out in accordance with the relevant guidelines and regulations. We prospectively enrolled 34 consecutive patients between October, 2013 and August, 2016. The inclusion criteria were as follows: (i) histologically confirmed cervical cancer, (ii) locally advanced tumor stages IB2 to IVA according to the International Federation of Gynecology and Obstetrics (FIGO) classification, (iii) undergoing CCRT in our institution and no treatment performed before, (iv) complete acquisition of MR examination for 4 times in the same 3.0-T MR scanner, (v) minimum follow-up period of 15 months after CCRT in patients without recurrence. Patients who quit or suspended therapy (n = 3) or insufficient image quality (n = 2) were considered not eligible for the study. Flowchart of the study population was shown in Fig. 4. Patient characteristics are detailed in Table 3.

**Table 3:** Patient characteristics (n = 34).

Characteristics	No. of patients (Percentages)
Age (years)	
Mean, range	52, 27 to 76
FIGO stage	
II	24 (70.6%)
III	8 (23.5%)
IV	2 (5.9%)
Pathology	
Squamous cell carcinoma	34 (100%)
Adenocarcinoma	0
MR Lymph node status	
Postive	18 (52.9%)
Negative	16 (47.1%)

Note: FIGO = the International Federation of Gynecology and Obstetrics; MR = magnetic resonance.

All the patients were scheduled to undergo 5-weeks external beam radiation therapy (EBRT) followed by 3-weeks intracavitary brachytherapy (ICBT). EBRT was delivered to the whole pelvis at 1.8–2.0 Gy daily, 5 days a week, with a total dose of 45–50 Gy. From the last week of EBRT, ICBT was given to point A (2 cm above the distal end of the lowest cervix and 2 cm lateral to the midline) at a fraction dose of 5 Gy, twice a week, with a total dose of 30–40 Gy. The total radiation time was within 8 weeks. Six cycles of weekly nedaplatin or four cycles of bi-weekly nedaplatin plus paclitaxel/docetaxel was given concomitantly. Adjustment of the therapeutic regimens was varied according to the health condition of individual patient.

### Clinical follow-up

Patients were evaluated posttherapy 1 month, 3 months and afterwards every 6 months until recurrence or last contact. Recurrence was defined as presence of histologically proven recurrence or progression of the primary tumor in the cervix, uterus or pelvis. Death from cervical cancer was also classified as recurrence group. Median follow-up of nonrecurrence patients were 31 months (range, 16–43 months).

### MR acquisitions

MR examinations were performed before CCRT, at early stage of CCRT (2 and 4 weeks after CCRT initiated) and one month after CCRT was completed. All the examinations were performed with the same 3.0 T MR scanner (Ingenia 3.0 T, Philips Healthcare, Best, The Netherlands) with a 16-channel torso phased array body coil. The imaging sequences included: (i) axial high-resolution T2-weighted turbo spin-echo sequence (TR = 4,500 ms, TE = 90 ms, matrix size = 308 × 402, FOV = 20 cm × 24 cm, slice thickness = 4 mm, intersection gap = 0.5 mm, NSA = 1), (ii) sagittal T2W TSE sequence (TR = 4500 ms, TE = 90 ms, matrix size = 480 × 354, FOV = 20 × 24 cm, slice thickness = 4 mm, intersection gap = 0.5 mm, NSA = 1), (iii) axial DW imaging with a free breathing spin-echo echo-planner-imaging sequence (TR = 3523–6000 ms, TE = shortest ms, matrix size = 132 × 157, FOV = 24 cm × 24 cm, slice thickness = 4 mm, intersection gap = 1 mm, NSA = 2, b value = 0 and 800 s/mm^2^). The MRI protocol was kept identical each time. No intravenous contrast medium was administered.

### Radiomic pipeline

The entire radiomic feature extraction was performed using the Imaging Biomarker Explorer (IBEX) software^27^. Pre-, mid- and post-treatment MRIs were analyzed by 2 radiologists (J.H. and Z.Z.), with 7 and 9 years’ experience in gynecological imaging, respectively), both were blinded to the results of patients’ outcomes. The regions of interest (ROIs) were drawn manually using the T2WI and DWI images on each slice covering the whole tumor. ROIs were placed on the slightly high signal intensity region on T2WI images and the high signal intensity region on DWI (b-value of 800 s/mm^2^) images and then copied to ADC maps. If no tumor signals were noted on post-CCRT images, then the ROIs were placed on the latest former tumor region. Next, 7 categories of different texture feature sets were extracted from the pre-, mid- and post-treatment T2WI and ADC data with manually delineated ROIs: (i) Gradient Orient Histogram (GOH) (ii) Gray-Level Co-occurrence Matrix (GLCM) from image inside the binary mask in 2.5D in 4 directions, GLCM × 25 (iii) GLCM from image inside the binary mask in 3D in 13 unique directions, GLCM × 3 (iv) Gray-Level Run Length Matrix (GLRLM) from image inside the binary mask in 2.5D in 0 and 90 degree, GLRLM × 25 (v) Intensity Direct (ID) (vi) Intensity Histogram Gauss Fit (IHGF) (vii) Neighbor Intensity Difference Matrix (NIDM) from image inside the binary mask and the neighborhood is in 2D, NIDM × 2. 713 textural parameters were extracted including 46 from GOH, 264 from GLCM × 25, 312 from GLCM × 3, 33 from GLRLM, 50 from ID, 3 from IHGF, 5 from NIDM × 2. The detailed texture features are briefly outlined in Table 4. Some of the higher order features’ names such as “RLN” or “LRHGE” sound hard to understand, the mathematical definition of those features can be found in the works of Haralick *et al*., Tang *et al*., Soh *et al*. and Amadasun *et al*.^28–31^.

**Table 4:** Texture features and abbreviations.

GOH	GLCM (×25; ×3)	GLRLM	ID	IHGF	NIDM
InterQuartileRange	AutoCorrelation	**GrayLevelNonuniformity (GLN)**	**Energy**	**GaussAmplitude**	Busyness
Kurtosis	ClusterProminence	HighGrayLevelRunEmpha (HGLRE)	EnergyNorm	**HistArea**	Coarseness
MeanAbsoluteDeviation	ClusterShade	LongRunEmphasis (LRE)	GlobalEntropy	**NumberOfGauss**	Complexity
MedianAbsoluteDeviation	ClusterTendendcy	**LongRunHighGrayLevelEmpha (LRHGE)**	GlobalMax/Mean/Median/Std		**Contrast**
**Percentile (5th, 65th)**	**Contrast (×3)**	**LongRunLowGrayLevelEmpha (LRLGE)**	GlobalUniformity		TextureStrength
**PercentileArea (40th)**	**Correlation (×25; ×3)**	LowGrayLevelRunEmpha (LGLRE)	InterOuartileRange		
Quantile	DifferenceEntropy	**RunLengthNonuniformity (RLN)**	Kurtosis		
**Range**	Dissimilarity	RunPercentage (RP)	**LocalEntropy**Max/Mean/Median/**Std**		
**Skewness**	Energy	ShortRunEmphasis (SRE)	LocalRangeMax/Mean/Median/Std		
	Entropy	ShortRunHighGrayLevelEmpha (SRHGE)	LocalStdMax/Mean/Median/Std		
	Homogeneity	ShortRunLowGrayLevelEmpha (SRLGE)	MeanAbsoluteDeviation		
	InformationMeasureCorr		MedianAbsoluteDeviation		
	InverseDiffMomentNorm		Percentile		
	InverseDiffNorm		Quantile		
	InverseVariance		Range		
	MaxProbability		RootMeanSquare		
	SumAverage		**Skewness**		
	**SumEntropy (×3)**		Variance		
	SumVariance				
	Variance				

Note: features in bold are the selected 20 textural parameters for further processing.

### Feature selection methods

Before establishing a prognostic model, feature filter is required mainly for three reasons: reducing the model’s training time, improving the robustness of the model and enhancing the model’s reliability and behavior. The chosen parameters should be reproducible, show high degree of differentiation and low redundancy. To analyze the reproducibility, the parameters were repeatedly measured at an interval of 6 weeks using pretreatment T2WI images. The concordance correlation coefficient (CCC) were used to evaluate the consistency of texture parameters extracted from two different measurements. We found the vast majority of features can meet high enough reproducibility with CCC value not lower than 0.9. A metric named dynamic range (DR) not lower than 0.9 implied that the feature had a large dynamic range^32^. Texture parameters with a CCC value ≥ 0.9 and a DR value ≥ 0.9 were extracted. Redundancy was assessed by computing interfeature correlation coefficient using R package corrgram. The features were grouped on the basis of the Pearson correlation coefficient between them, we chose 0.8 as cutoff value for the Pearson correlation coefficient. In this subset, one representative that had the highest DR was picked. Using the above methods, 20 texture parameters were selected for further processing of the study (see details in Table 4).

### Statistical analyses

All statistical analyses were performed using R software version 3.4.3 and SPSS 22.0 software (SPSS Inc., Chicago, IL). Multivariate analysis of variance (MANOVA) was used to test the difference between the two groups of each feature at different time points along the course of disease. Feature variety trend was investigated with the 4-factor repeated measures ANOVA test. Binary logistic regression analysis (forward LR stepwise method) was used to construct multi-indicator models for prediction of recurrence. Receiver operating characteristic (ROC) analysis was performed to assess the predictive value of those models by calculating the areas under the ROC curve (AUCs) and the corresponding P values. P values of less than 0.05 were considered statistically significant. Comparisons between AUCs were performed by using MedCalc Statistical Software version 15.2.2 (MedCalc Software bvba, Ostend, Belgium; http://www.medcalc.org; 2015). The support vector machine (SVM) classifier was used for supervised learning on T2, ADC and T2 + ADC textural parameters, respectively. The stratified k-fold cross-validation (CV) was then used as the internal validation to evaluate the accuracy, sensitivity and specificity of the classification. The stratified approach was chosen in order to ensure that both the recurrence and nonrecurrence types were represented in the validation folds. This process was repeated 100 times to include all the possible ways of obtaining such a partition in our dataset, and the results were then averaged.

### Data availability

The datasets generated during and/or analyzed during the current study are available from the corresponding author on reasonable request.
